# Association between triglyceride glucose index and all-cause mortality in patients with cerebrovascular disease: a retrospective study

**DOI:** 10.1186/s13098-023-01243-2

**Published:** 2024-01-03

**Authors:** Yong’An Jiang, Peng Chen, YangYang Zhao, JiaHong Cai, JiaWei Liang, ShiQi Cheng, Yan Zhang

**Affiliations:** 1 Department of Neurosurgery, The Second Affiliated Hospital, Jiangxi Medical College, Nanchang University, Nanchang, Jiangxi 330006 P. R. China; 2 Nanchang University, Nanchang, Jiangxi 330008 P. R. China

**Keywords:** Cerebrovascular Disease (CVD), Medical Information Mart for Intensive Care IV (MIMIC-IV), Triglyceride glucose index (TyG), Mortality

## Abstract

**Background:**

Triglyceride glucose (TyG) is associated with stroke, atherosclerosis, and adverse clinical outcomes. However, its correlation with cerebrovascular disease (CVD) mortality remains unclear. This study aimed to investigate the relationship between TyG index and mortality in patients with CVD.

**Methods:**

Patient data sourced from the Medical Information Mart for Intensive Care -IV database were categorized based on TyG quartiles. Kaplan–Meier survival analysis was used to estimate survival disparities among the TyG subgroups. Cox proportional risk modeling was used to examine the association between the TyG index and mortality. Generalized summation models were applied to fit the smoothed curves. log-likelihood ratio test were used to analyze the non-linear relationship.

**Results:**

The study comprised 1,965 patients (50.18% were male). The 28-day and 90-day mortality rates were 20.10% and 24.48%, respectively. The TyG index exhibited a linear relationship with the 28-day mortality (Hazards ratio (HR), 1.16; 95% confidence interval (CI), 0.99–1.36) and the 90-day mortality (HR, 1.18; 95% CI, 1.02–1.37). In the TyG Q4 group, each 1 mg/dl increase was linked to a 35% rise in the risk of 28-day mortality and a 38% increase in the risk of 90-day mortality. Subgroup analyses highlighted a more substantial association between TyG index and 90-day mortality in the diabetic group.

**Conclusion:**

Our findings underscore the positive association between TyG and the 28- and 90-day mortality rates in patients with CVD. This insight may prove pivotal for identifying at-risk populations and enhancing risk prediction in the clinical management of CVD.

**Supplementary Information:**

The online version contains supplementary material available at 10.1186/s13098-023-01243-2.

## Background

Cerebrovascular disease (CVD) is a significant contributor to the global disease burden and is characterized by a range of disorders such as vascular lumen occlusion, vascular rupture, malformations, vessel wall damage, altered permeability, and other cerebral dysfunctions, both localized and diffuse. These changes, induced by various cerebrovascular disorders, lead to pathological alterations in the cerebral vasculature [1–4]. Conditions such as stroke [5], non-traumatic cerebral hemorrhage [6], cerebral aneurysm [7], cerebral artery embolism [8], and cerebral hemorrhage [9] result in substantial neuromotor deficits and diminished quality of life and often result in elevated mortality rates.

Insulin resistance (IR) denotes a diminished response to insulin regulation, reflecting disrupted insulin and glucose metabolism. Numerous studies have established a close connection between IR and the development of cardiovascular and cerebrovascular diseases, which significantly affect disease prognosis [10, 11]. The triglyceride glucose (TyG) index, an indicator of IR, has been linked to cardiovascular events and is closely associated with atherosclerosis, coronary artery disease, and ischemic stroke [12–14]. However, there are conflicting results regarding its correlation with intracranial hemorrhage [15], cardiovascular disease [16], and CVD [17].

This study retrospectively analyzed the demographics, comorbidities, and experimental indicators of patients with CVD using the Medical Information Mart for Intensive Care IV (MIMIC-IV) to thoroughly assess the association between TyG and 28- and 90-day mortality rates in patients with CVD. This study aimed to develop new clinical treatment strategies to improve the prognosis of patients with CVD.

## Methods

### Inclusion and exclusion of patients with CVD in the MIMIC-IV database

The MIMIC-IV database is a valuable resource for clinical researchers that offers de-identified patient data free of charge. Established in 2003 through collaborative efforts by the Laboratory of Computational Physiology at the Massachusetts Institute of Technology (MIT), the Beth Israel Deaconess Medical Center (BIDMC) at Harvard Medical School (HMS), and Philips Healthcare, this database received funding from the National Institutes of Health (NIH) in the United States. One of the authors (JYA) gained access to this database (Record ID: 58,572,169) on completing the NIH online course.

Ethical considerations were paramount to our study. The project was approved by the Institutional Review Boards of both MIT and BIDMC, and informed consent was waived.

For our research, patients with CVD were meticulously screened based on the ICD-9 and ICD-10 codes. Individuals aged > 18 years were included in this study. To ensure accuracy, only the first admissions were considered, and patients with ICU stays lasting < 24 h were excluded. Additionally, individuals lacking crucial triglyceride and glucose data during initial admission were excluded.

**Fig. 1:**
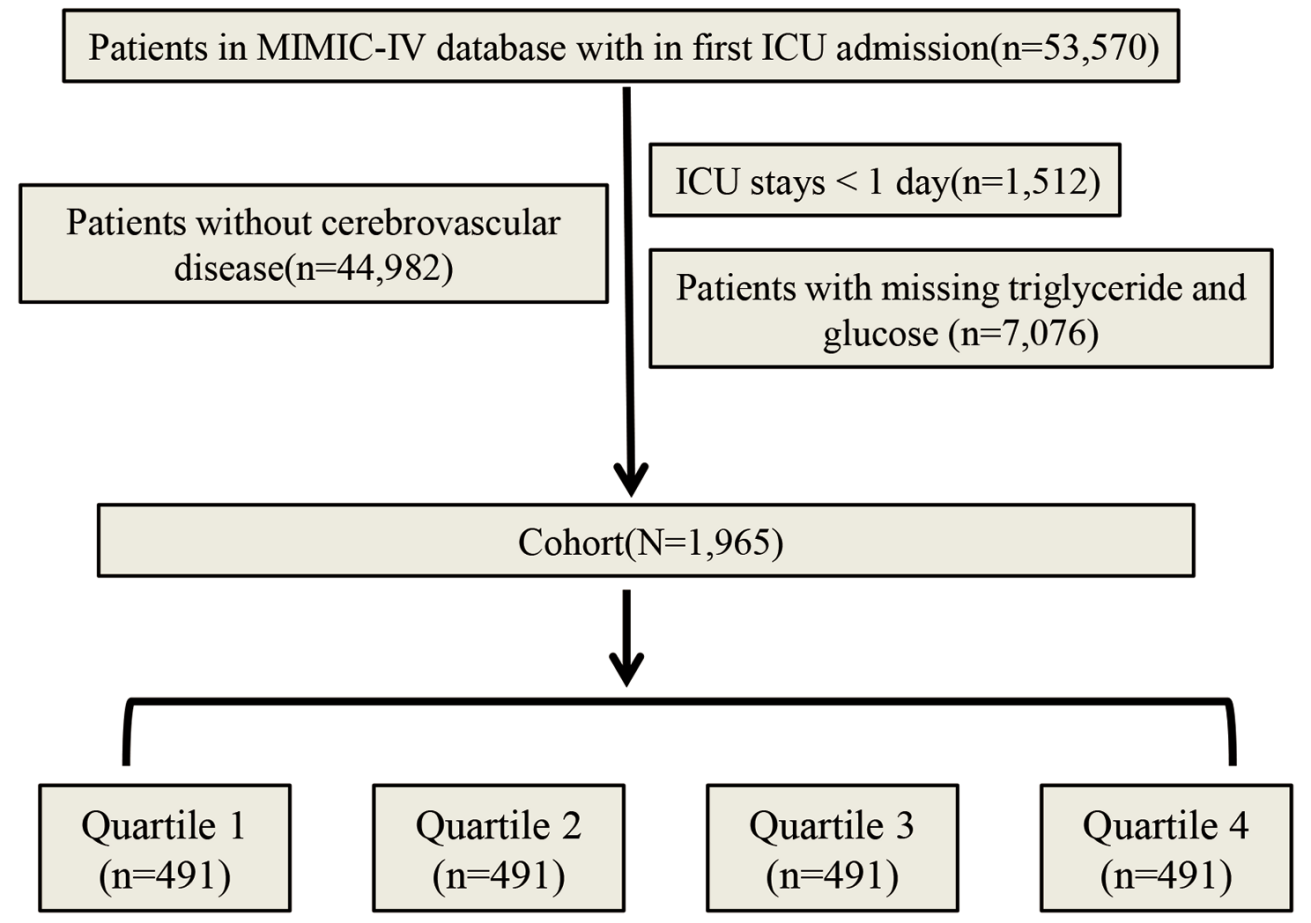
Flowchart of the study

Advanced tools were applied to extract the necessary clinical information. Software applications such as PostgresSQL (version 13.7.2) and Navicat Premium (version 16) facilitated the execution of Structured Query Language (SQL) commands, ensuring precise data retrieval. The flowchart of this study is presented in Fig. 1.

### Extraction of clinical information of patients and data processing

Patient’s clinical information was meticulously extracted using the SQL language and contained vital details, such as sex, admission age (years), weight (kg), and ethnicity. In addition, a comprehensive array of laboratory indicators were obtained, including international normalized ratio (INR), glucose (mg/dL), triglycerides (mg/dL), prothrombin time (PT, s), partial thromboplastin time (PTT, s), white blood cell count (WBC, K/µl), chloride (mg/dL), red blood cell (RBC, m/µl), platelet (K/µl), heart rate (beats/min), respiratory rate (bpm), systolic blood pressure (SBP, mmHg), and diastolic blood pressure (DBP, mmHg). Furthermore, the presence of comorbidities, including myocardial infarction, congestive heart failure, peripheral vascular disease, dementia, paraplegia, renal disease, atrial fibrillation, and diabetes, was meticulously recorded. Disease severity was assessed using the Sequential Organ Failure Assessment (SOFA) score. Additionally, information regarding drug therapy, specifically antihyperglycemic (carbose, miglitol, saxagliptin, metformin, glimepiride, glipizide, repaglinide, rosiglitazone, and pioglitazone) and antihyperlipidemic (atorvastatin, fluvastatin, rosuvastatin, pitavastatin, simvastatin, lovastatin, mycostatin, pravastatin, fenofibrate, gemfibrozil, and fenofibrate) drugs was included.

For data accuracy, the initial recorded value was considered when a variable was logged more than once within the preceding 24 h. The follow-up period, which is crucial for tracking patient progress, commenced on the admission date and concluded upon the occurrence of a specific endpoint of interest.

### Calculation of triglyceride glucose (TyG) index

The TyG index, a pivotal metric, was computed using the following formula: Ln (Triglycerides [mg/dl] × Glucose [mg/dl]/2). This index is a fundamental parameter in the evaluation process.

### Management of missing data

A meticulous approach was adopted to handle missing data in the MIMIC dataset. Variables with > 20% prevalence of missing values were excluded from the study to ensure analytical integrity. For variables with < 20% missing values, a robust methodology employing multiple interpolation techniques was employed, carefully filling in the gaps while preserving data coherence and reliability (Table S1).

### Clinical outcome

The primary outcome was the 28-day mortality. Secondary outcomes were 90-day mortality and length of hospital stay.

### Statistical analysis

In the present study, our analysis focused on patients diagnosed with CVD, categorized into four groups (Q1 [7.21–8.40] mg/dl, Q2 [8.40–8.80] mg/dl, Q3 [8.80–9.24] mg/dl, and Q4 [9.24–12.96] mg/dl) based on quartiles of the recorded TyG index. Continuous variables are presented as mean ± standard deviation (SD) or median (interquartile range, IQR), while categorical variables are expressed as numbers and frequency percentiles (%). Fisher’s exact or Pearson’s chi-square tests were used to compare categorical variables between the groups. The Mann–Whitney U or Kruskal–Wallis tests were used to assess normal distribution,.

Initially, the Kaplan–Meier survival analysis was employed to evaluate the incidence of various outcomes within the different TyG subgroups. Differences in components were estimated using log-rank tests. Prior to applying the Cox proportional risk model, we validated the Cox proportional risk hypothesis and calculated hazard ratios (HRs) and 95% confidence intervals (CIs) for the association between the TyG index and CVD mortality. Covariates were adjusted for multiple models to avoid multicollinearity (exclusion of variables with a variance inflation factor > 10). Four multivariable-adjusted models were constructed: Model I involved no adjustments; Model II was adjusted for demographic indicators (sex, admission age, weight, and ethnicity); Model III included Model I and INR, PT, PTT, WBC, chloride, RBC, platelet, heart rate, respiratory rate, SBP, DBP, myocardial infarction, congestive heart failure, peripheral vascular disease, dementia, paraplegia, renal disease, atrial fibrillation, diabetes, and SOFA; and Model IV included Model III along with antihyperglycemic and antihyperlipidemic drugs. Both continuous TyG indices and quartile-based TyG indices were analyzed, and the p-value for trend was derived from the TyG quartiles.

Generalized summation models were applied to explore nonlinear relationships by adjusting for the aforementioned models. log-likelihood ratio test were used to analyze the non-linear relationship. HRs and 95% CIs were calculated for the different segments.

Furthermore, subgroup analyses were performed to investigate heterogeneity in the TyG index and 28- and 90-day mortality rates across various subgroups. Stratified analyses were carried out based on sex (male and female), admission age (≤ 60 and > 60 years), presence of myocardial infarction (No and Yes), congestive heart failure (No and Yes), peripheral vascular disease (No and Yes), dementia (No and Yes), paraplegia (No and Yes), renal disease (No and Yes), atrial fibrillation (No and Yes), and diabetes (No and Yes). Interactions between the variables were examined using the likelihood ratio test.

All statistical analyses were conducted using R software (v.4.2.3; R Basis for Statistical Computing, Vienna, Austria), and a significance level of *p* < 0.05 was considered statistically significant.

## Results

In our retrospective analysis, we examined a cohort of 1,965 patients diagnosed with CVD based on stringent inclusion and exclusion criteria. The average age of the participants was 71.97 (IQR: 60.55–82.76) years. Among these individuals, 986 (50.18%) were male, and 979 (49.82%) were female. The TyG index, a key parameter in our study, was 8.80 (IQR: 8.40–9.24). Notably, our investigation revealed 395 (20.10%) ICU deaths within 28 days of follow-up and 481 (24.48%) ICU deaths within 90 days of follow-up (Table 1).

**Table 1:** Baseline characteristics of TyG quartile-based grouping

TyG quartile	Overall	Q1	Q2	Q3	Q4	*p*-value
**N**	1,965	491	491	491	492	
**Admission age (years, IQR)**	71.97 (60.55–82.76)	76.75 (63.85–86.55)	75.45 (63.11–84.06)	71.78 (60.01–82.23)	67.07 (56.45–76.87)	< 0.001
**Weight (Kg, IQR)**	77.00 (64.00–91.00)	71.80 (61.10–82.40)	75.30 (63.00–89.80)	78.50 (65.60–94.10)	83.00 (68.40–99.50)	< 0.001
**INR (IQR)**	1.10 (1.00–1.20)	1.10 (1.00–1.20)	1.10 (1.00–1.20)	1.10 (1.00–1.20)	1.10 (1.00–1.20)	0.363
**PT (Second, IQR)**	12.20 (11.30–13.70)	12.30 (11.40–13.70)	12.20 (11.35–13.95)	12.10 (11.30–13.50)	12.20 (11.30–13.80)	0.579
**PTT (Second, IQR)**	28.00 (25.50–31.50)	28.50 (25.80–31.70)	28.00 (25.55–31.80)	27.60 (25.50–31.00)	27.70 (24.90–31.40)	0.045
**WBC (K/ul, IQR)**	9.60 (7.50–12.70)	8.60 (6.70–10.60)	9.50 (7.40–12.30)	10.20 (8.25–13.30)	10.90 (8.20–14.70)	< 0.001
**Glucose (mg/dL, IQR)**	123.00 (103.00–157.00)	102.00 (91.00–119.50)	116.00 (101.50–137.00)	131.00 (111.00–159.00)	170.00 (133.00–235.25)	< 0.001
**Chloride (mg/dL, IQR)**	103.00 (100.00–106.00)	104.00 (101.00–107.00)	104.00 (101.00–106.00)	103.00 (100.00–106.00)	102.00 (99.00–105.00)	< 0.001
**Triglycerides (mg/dL, IQR)**	104.00 (76.00–147.00)	66.00 (55.00–77.50)	93.00 (79.00–109.00)	122.00 (101.00–148.00)	186.50 (144.00–258.25)	< 0.001
**TyG (IQR)**	8.80 (8.40–9.24)	8.16 (8.00–8.30)	8.60 (8.52–8.70)	9.00 (8.89–9.11)	9.63 (9.40–10.02)	< 0.001
**RBC (m/ul, IQR)**	4.26 (3.80–4.70)	4.17 (3.77–4.56)	4.26 (3.75–4.66)	4.33 (3.85–4.75)	4.34 (3.79–4.78)	< 0.001
**Platelet (K/uL, IQR)**	217.00 (171.00–271.00)	210.00 (161.50–259.00)	214.00 (177.00–269.00)	218.00 (174.50–271.50)	222.00 (173.75–286.00)	0.026
**Heart rate (beats/min, IQR)**	80.00 (69.00–92.00)	78.00 (67.00–89.00)	80.00 (70.00–92.00)	79.00 (69.00–91.00)	84.00 (72.00–96.00)	< 0.001
**Respiratory rate (bpm, IQR)**	18.00 (15.00–22.00)	18.00 (15.00–21.00)	18.00 (15.00–21.00)	18.00 (16.00–22.00)	19.00 (16.00–22.00)	0.009
**SBP (mmHg, IQR)**	141.00 (125.00–157.00)	140.00 (125.00–156.00)	142.00 (126.50–156.00)	142.00 (127.00–158.00)	142.00 (124.00–157.00)	0.558
**DBP (mmHg, IQR)**	77.00 (66.00–89.00)	77.00 (66.00–87.00)	77.00 (67.00– 88.00)	76.00 (66.00–89.00)	77.50 (66.75–91.00)	0.645
**SOFA (Score, IQR)**	3.00 (2.00–5.00)	3.00 (2.00–4.00)	3.00 (2.00–5.00)	3.00 (2.00–5.00)	4.00 (2.00–6.00)	< 0.001
**Sex (n,%)**						0.429
**Male**	986 (50.18%)	260 (52.95%)	241 (49.08%)	249 (50.71%)	236 (47.97%)	
**Female**	979 (49.82%)	231 (47.05%)	250 (50.92%)	242 (49.29%)	256 (52.03%)	
**Ethnicity (n,%)**						0.155
**Black**	1208 (61.48%)	313 (63.75%)	311 (63.34%)	289 (58.86%)	295 (59.96%)	
**White**	184 (9.36%)	53 (10.79%)	44 (8.96%)	39 (7.94%)	48 (9.76%)	
**Others**	573 (29.16%)	125 (25.46%)	136 (27.70%)	163 (33.20%)	149 (30.28%)	
**Myocardial infarction (n,%)**						0.028
**No**	1704 (86.72%)	443 (90.22%)	429 (87.37%)	415 (84.52%)	417 (84.76%)	
**Yes**	261 (13.28%)	48 (9.78%)	62 (12.63%)	76 (15.48%)	75 (15.24%)	
**Congestive heart failure (n,%)**						0.281
**No**	1578 (80.31%)	399 (81.26%)	395 (80.45%)	403 (82.08%)	381 (77.44%)	
**Yes**	387 (19.69%)	92 (18.74%)	96 (19.55%)	88 (17.92%)	111 (22.56%)	
**Peripheral vascular disease (n,%)**						0.783
**No**	1779 (90.53%)	443 (90.22%)	440 (89.61%)	449 (91.45%)	447 (90.85%)	
**Yes**	186 (9.47%)	48 (9.78%)	51 (10.39%)	42 (8.55%)	45 (9.15%)	
**Dementia (n,%)**						0.015
**No**	1837 (93.49%)	446 (90.84%)	457 (93.08%)	463 (94.30%)	471 (95.73%)	
**Yes**	128 (6.51%)	45 (9.16%)	34 (6.92%)	28 (5.70%)	21 (4.27%)	
**Paraplegia (n,%)**						0.204
**No**	916 (46.62%)	224 (45.62%)	214 (43.58%)	231 (47.05%)	247 (50.20%)	
**Yes**	1049 (53.38%)	267 (54.38%)	277 (56.42%)	260 (52.95%)	245 (49.80%)	
**Renal disease (n,%)**						0.003
**No**	1673 (85.14%)	437 (89.00%)	421 (85.74%)	418 (85.13%)	397 (80.69%)	
**Yes**	292 (14.86%)	54 (11.00%)	70 (14.26%)	73 (14.87%)	95 (19.31%)	
**Atrial fibrillation (n,%)**						< 0.001
**No**	1255 (63.87%)	296 (60.29%)	283 (57.64%)	326 (66.40%)	350 (71.14%)	
**Yes**	710 (36.13%)	195 (39.71%)	208 (42.36%)	165 (33.60%)	142 (28.86%)	
**Diabetes (n,%)**						< 0.001
**No**	1371 (69.77%)	439 (89.41%)	396 (80.65%)	326 (66.40%)	210 (42.68%)	
**Yes**	594 (30.23%)	52 (10.59%)	95 (19.35%)	165 (33.60%)	282 (57.32%)	
**Antihyperglycemic (n,%)**						0.003
**No**	1950 (99.24%)	486 (98.98%)	490 (99.80%)	491 (100.00%)	483 (98.17%)	
**Yes**	15 (0.76%)	5 (1.02%)	1 (0.20%)	0 (0.00%)	9 (1.83%)	
**Antihyperlipidemic (n,%)**						0.900
**No**	1197 (60.92%)	302 (61.51%)	303 (61.71%)	299 (60.90%)	293 (59.55%)	
**Yes**	768 (39.08%)	189 (38.49%)	188 (38.29%)	192 (39.10%)	199 (40.45%)	

TyG, triglyceride glucose index; INR, international normalized ratio; PT, prothrombin time; PTT, partial thromboplastin time; WBC, white blood cell; RBC, red blood cell; SBP, systolic blood pressure; DBP, diastolic blood pressure; SOFA, sequential organ failure assessment; IQR, interquartile range. TyG index quartiles: Q1 (7.21–8.40) mg/dl, Q2 (8.40–8.80) mg/dl, Q3 (8.80–9.24) mg/dl, and Q4 (9.24–12.96) mg/dl

### Baseline characteristics

We systematically categorized all baseline characteristics of patients with CVD based on the TyG index quartiles. Patients with a higher TyG index were typically younger and had a higher body weight and elevated levels of WBC, glucose, and triglycerides than those with a lower TyG index. Additionally, they exhibited increased RBC and platelet counts, higher heart and respiratory rates, and elevated SBP and DBP. Notably, these patients also had lower chloride levels.

Regarding the duration of hospitalization, our findings indicated that patients with a higher TyG index had a prolonged in-hospital stay (6.27 vs. 6.70 vs. 6.97 vs. 7.81 days, *p* < 0.001) and a longer ICU stay (2.77 vs. 3.00 vs. 3.27 vs. 3.21 days, *p* = 0.003) in comparison to their counterparts. Moreover, the incidence of adverse events, both at the 28-day (16.70 vs. 21.38 vs. 19.55 vs. 22.76%, *p* = 0.098) and 90-day follow-ups (20.37 vs. 26.27 vs. 23.22 vs. 28.05%, *p* = 0.027) was notably higher among patients with a higher TyG index (Table S2).

In terms of complications, individuals with an elevated TyG index were significantly more prone to developing diabetes (10.59 vs. 19.35 vs. 33.60 vs. 57.31%, *p* < 0.001) and renal disease (11.00 vs. 14.26 vs. 14.87 vs. 19.31%, *p* = 0.003). These patients were less likely to develop dementia (9.16 vs. 6.92 vs. 5.70 vs. 4.27%, *p* = 0.015).

Furthermore, our analysis of therapeutic interventions revealed that patients with a higher TyG index were more likely to receive antihyperlipidemic drugs (38.49 vs. 38.29 vs. 39.10 vs. 40.45%, *p* = 0.900) (Table 1).

### Mortality analysis based on TyG quartiles

**Fig. 2:**
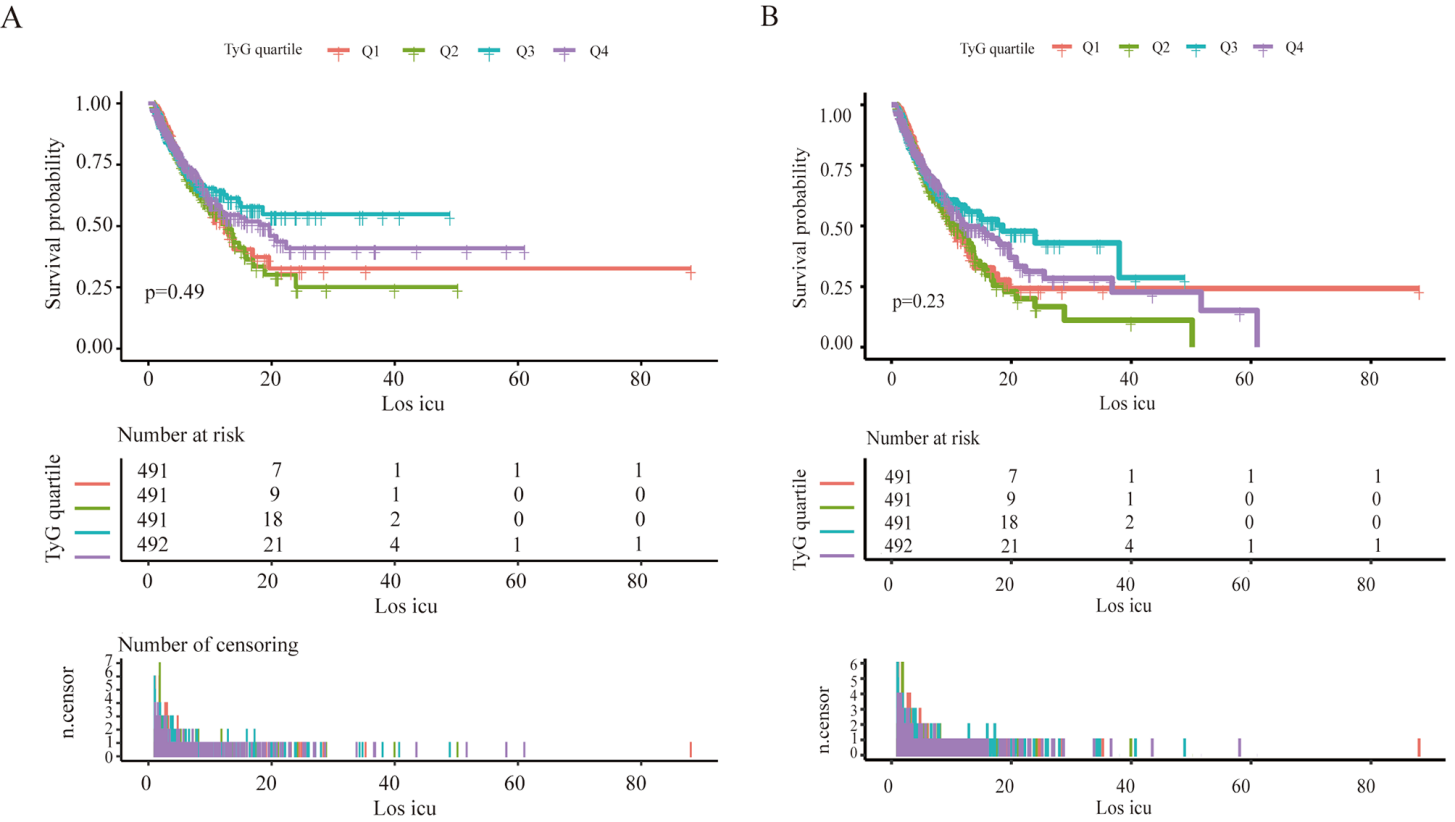
Kaplan–Meier survival analysis curve for 28- and 90-day mortality. **(A)** 28-day mortality; **(B)** 90-day mortality. TyG, triglyceride glucose index. TyG index quartiles: Q1 (7.21–8.40) mg/dl, Q2 (8.40–8.80) mg/dl, Q3 (8.80–9.24) mg/dl, and Q4 (9.24–12.96) mg/dl

Survival analyses using Kaplan–Meier curves were conducted to evaluate the 28- and 90-day mortality across various TyG quartile groups. However, no significant differences were observed in the 28- and 90-day mortality rates among the quartile groups (log-rank *p* = 0.49 and *p* = 0.23, respectively) (Fig. 2). TyG appeared to be superior in predicting the 28-day mortality compared to the use of triglycerides or glucose alone (area under the curve [AUC] = 0.50, specificity = 0.72, sensitivity = 0.31) but performed poorly for 90-day mortality (Table S3).

### Association between TyG index and 28- and 90-day mortality rates

**Fig. 3:**
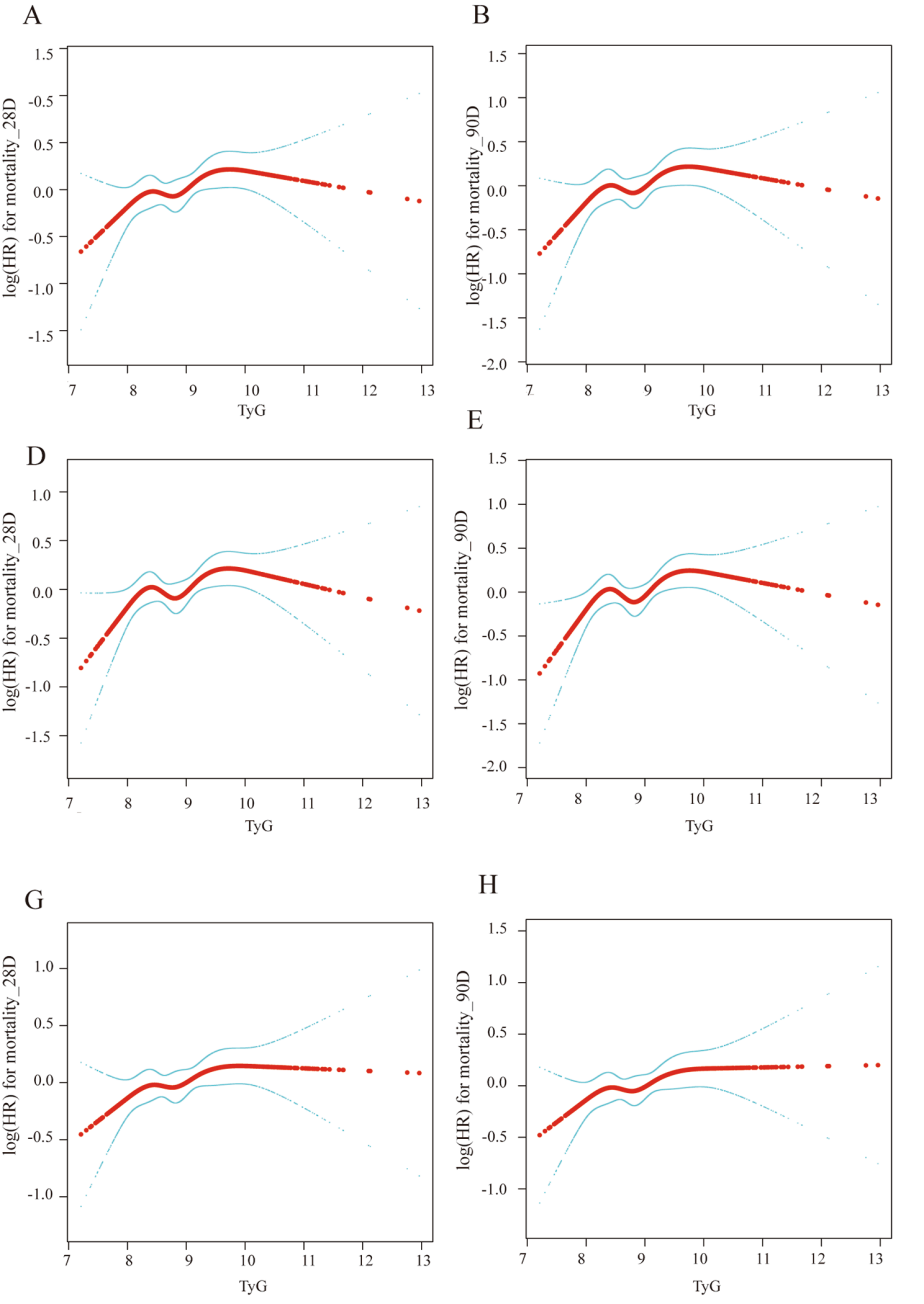
Smoothed curve fitting analysis based on generalized additive models (different adjustment models). Smoothed plot of TyG index vs. 28-day mortality (**A**) and 90-day mortality (**B**), adjusted only for sex, admission age, weight, and ethnicity. TyG index vs. 28-day mortality (**C**) and 90-day mortality (**D**), adjusted only for sex, admission age, weight, and ethnicity, INR, PT, PTT, WBC, Chloride, RBC, platelet, heart rate, respiratory rate, SBP, DBP, myocardial infarction, congestive heart failure, peripheral vascular disease, dementia, paraplegia, renal disease, atrial fibrillation, diabetes, and SOFA. TyG index vs. 28-day mortality (**E)** and 90-day mortality (**F**), adjusted only for sex, admission age, weight, and ethnicity, INR, PT, PTT, WBC, chloride, RBC, platelet, hart rate, respiratory rate, SBP, DBP, myocardial infarction, congestive heart failure, peripheral vascular disease, dementia, paraplegia, renal disease, atrial fibrillation, diabetes, SOFA, antihyperglycemic, and antihyperlipidemic drugs

Our study employed a generalized additive model to create a smooth curve representing the TyG index and mortality relationship. This curve exhibited an M-shaped pattern, indicating that the first half of the mortality rate increased as the TyG index rose, while the second half decreased beyond a certain turning point. The turning point of this curve was identified using a segmented regression model and the maximum likelihood method, yielding a TyG index value of 8.8 mg/dl (Fig. 3).

Our results revealed that the TyG index, when treated as a continuous variable, exhibited a linear association with 28- and 90-day mortality rates (*p* for the log-likelihood ratio > 0.05 for both). Specifically, every 1 mg/dl increase in the TyG index corresponded to a 16% increased risk of death within 28 days (*p* = 0.0656) and an 18% increased risk of death within 90 days (*p* = 0.0231). This relationship held true across the various adjusted models, as evidenced by the unadjusted Model I (HR, 0.94; 95% CI, 0.82–1.05; *p* = 0.2306) and Model IV (HR, 1.18; 95% CI, 1.02–1.37; *p* = 0.0231). The association of TyG with 90-day mortality was consistent with that for 28-day mortality (Table 2).

The above analysis underscores the linear correlation between the TyG index and the mortality risk. Furthermore, we extended our analysis to investigate the association between TyG level as a categorical variable and mortality. Following complete adjustment for laboratory parameters, complications, scores, and therapeutic agents in Model IV, TyG exhibited a correlation with the 90-day mortality, with Q1 as the reference group (Q2: HR, 1.16; 95% CI, 0.88–1.51; *p* = 0.2957; Q3: HR, 1.09; 95% CI, 0.82–1.45; *p* = 0.5447; Q4: HR, 1.38; 95% CI, 1.03–1.86; *p* = 0.0308; *p* for trend = 0.057). A similar trend was observed for TyG with respect to 28-day mortality in the fully adjusted Model IV (Table 2).

Our analysis indicates a positive association between the TyG index, treated as a continuous or categorical variable, and 28- and 90-day mortality rates.

**Table 2:** Cox proportional risk analysis for 28- and 90-day mortality

Exposure	Model I^a^ HR (95% CI) *p*	*p* for trend	Model II^b^ HR (95% CI) *p*	*p* for trend	Model III^c^ HR (95% CI) *p*	*p* for trend	Model IV^d^ HR (95% CI) *p*	*p* for trend
**Mortality_28d**								
**TyG (continuous per 1 unit)**	0.94 (0.82, 1.07) 0.3585		1.15 (1.00, 1.32) 0.0444		1.12 (0.97, 1.30) 0.1231		1.16 (0.99, 1.36) 0.0656	
**TyG quartile**		0.441		0.041		0.104		0.099
**Q1**	Ref		Ref		Ref		Ref	
**Q2**	1.12 (0.84, 1.50) 0.4424		1.20 (0.90, 1.60) 0.2201		1.14 (0.85, 1.52) 0.3998		1.14 (0.85, 1.54) 0.3883	
**Q3**	0.91 (0.67, 1.22) 0.5150		1.14 (0.85, 1.54) 0.3769		1.13 (0.83, 1.53) 0.4424		1.12 (0.82, 1.52) 0.4861	
**Q4**	0.96 (0.72, 1.28) 0.7799		1.40 (1.05, 1.89) 0.0243		1.31 (0.96, 1.78) 0.0880		1.35 (0.97, 1.88) 0.0706	
**Mortality_90d**								
**TyG (continuous per 1 unit)**	0.93 (0.82, 1.05) 0.2306		1.14 (1.01, 1.29) 0.0391		1.14 (1.00, 1.30) 0.0481		1.18 (1.02, 1.37) 0.0231	
**TyG quartile**		0.283		0.046		0.065		0.057
**Q1**	Ref		Ref		Ref		Ref	
**Q2**	1.13 (0.87, 1.47) 0.3473		1.20 (0.92, 1.56) 0.1684		1.16 (0.89, 1.52) 0.2738		1.16 (0.88, 1.51) 0.2957	
**Q3**	0.88 (0.67, 1.15) 0.3436		1.11 (0.84, 1.45) 0.4632		1.10 (0.84, 1.46) 0.4860		1.09 (0.82, 1.45) 0.5447	
**Q4**	0.94 (0.73, 1.22) 0.6635		1.37 (1.05, 1.79) 0.0219		1.34 (1.01, 1.77) 0.0390		1.38 (1.03, 1.86) 0.0308	

TyG, triglyceride glucose index; HR, hazard ratio; 95% CI, 95% confidence interval

^a^was unadjusted

^b^was adjusted for sex, admission age, weight, and ethnicity

^c^was adjusted for sex, admission age, weight, and ethnicity, INR, PT, PTT, WBC, chloride, RBC, platelet, heart rate, respiratory rate, SBP, DBP, myocardial infarction, congestive heart failure, peripheral vascular disease, dementia, paraplegia, renal disease, atrial fibrillation, diabetes, and soft tissue

^d^was adjusted for sex, admission age, weight, and ethnicity, INR, PT, PTT, WBC, chloride, RBC, platelet, heart rate, respiratory rate, SBP, DBP, myocardial infarction, congestive heart failure, peripheral vascular disease, dementia, paraplegia, renal disease, atrial fibrillation, diabetes, soft, antihyperglycemic, and antihyperlipidemic drugs

TyG index quartiles: Q1 (7.21–8.40) mg/dl, Q2 (8.40–8.80) mg/dl, Q3 (8.80–9.24) mg/dl, and Q4 (9.24–12.96) mg/dl

### Subgroup analysis

**Fig. 4:**
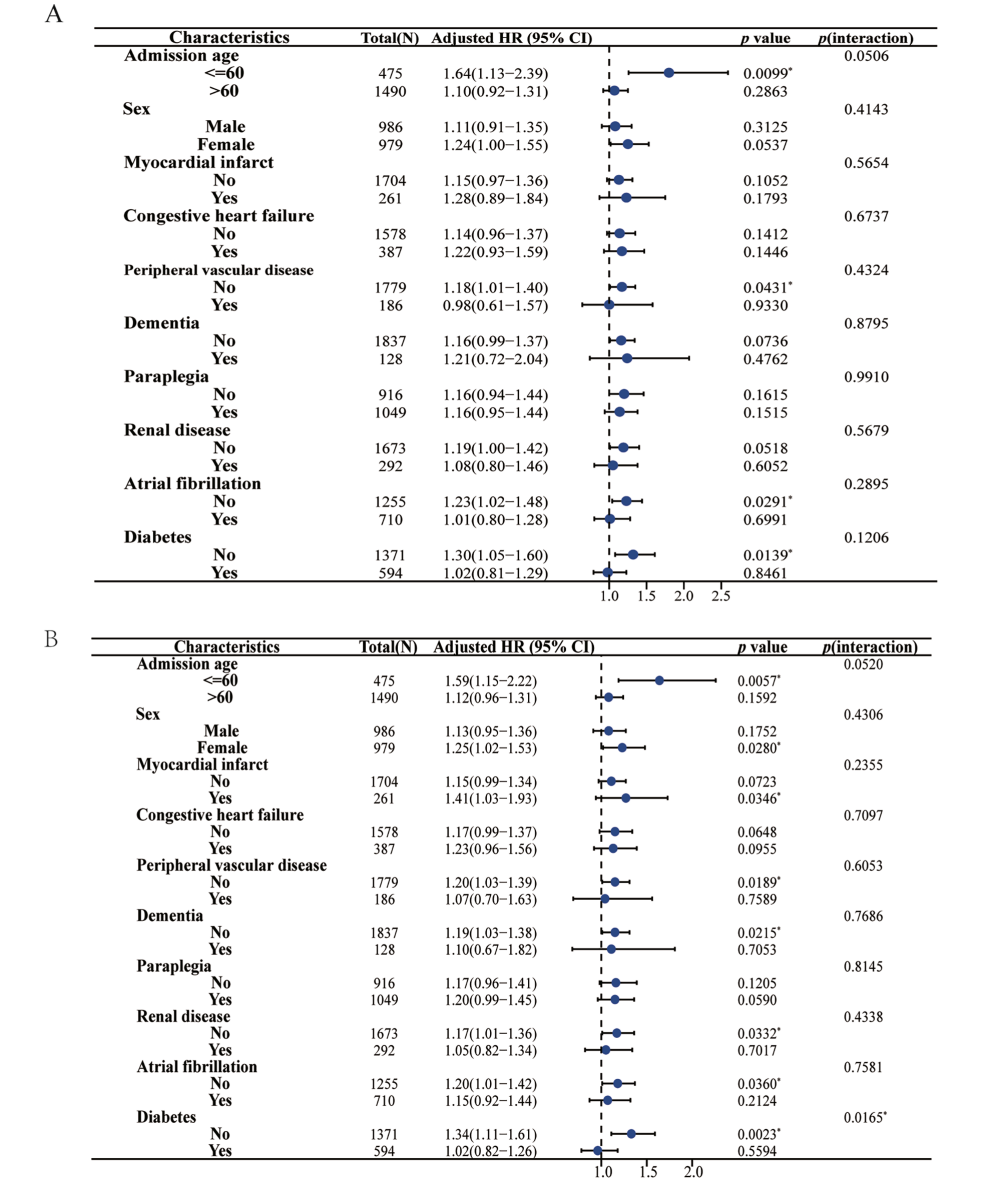
Subgroup analysis of TyG index and 28- and 90-day mortality. **A**. 28-day mortality; **B**. 90-day mortality. HR, hazard ratio; 95% CI, 95% confidence interval

A stratified analysis of the TyG index with 28- and 90-day mortality rates was performed based on potential modifying factors in the study population (age, sex, myocardial infarction, congestive heart failure, peripheral vascular disease, dementia, paraplegia, renal disease, atrial fibrillation, and diabetes). For age ≤ 60 years (HR, 1.59; 95% CI, 1.15–2.22; *p* = 0.0057), female sex (HR, 1.25; 95% CI, 1.02–1.53; *p* = 0.0280), myocardial infarction (HR, 1.41; 95% CI, 1.03–1.93; *p* = 0.0346), no peripheral vascular disease (HR, 1.20; 95% CI, 1.03–1.39; *p* = 0.0189), no renal disease (HR, 1.17; 95% CI, 1.01–1.36; *p* = 0.0332), and no atrial fibrillation (HR, 1.20; 95% CI, 1.01–1.42; *p* = 0.0360), TyG indices appeared to be more prominent with age and diabetes mellitus (with diabetes [no: HR, 1.34; 95% CI, 1.11–1.61; *p* = 0.0023; yes: HR, 1.02; 95% CI, 0.82–1.26; *p* = 0.5594; *p* for interaction = 0.0165]). Similar results were found in the stratified analyses of the TyG index versus 28-day mortality (Fig. 4).

### Sensitivity analyses

It is well known that antihyperglycemic and antihyperlipidemic drugs affect TyG metabolism. We performed a series of sensitivity analyses and found that TyG as a continuous and categorical variable showed similar results after excluding patients on antihyperglycemic and antihyperlipidemic drugs, respectively (Tables S3 and S4).

## Discussion

In this study, we investigated the intricate relationship between the TyG index and 28- and 90-day mortality rates associated with CVD. Through extensive retrospective analyses using the MIMIC database, we found a significant positive correlation between TyG index and mortality. Notably, as the TyG index increased, the risk of 28- and 90-day mortality rates escalated proportionally. This association persisted even after meticulous adjustments for all potential confounding variables, as substantiated by robust sensitivity analyses. This steadfast correlation underscores the pivotal role of the TyG index in clinical prevention, diagnosis, and treatment of CVD. Its utility lies not only in optimizing risk stratification for patients with CVD but also in guiding clinical decisions.

Our findings are supported by broader evidence from diverse studies. A meta-analysis revealed that a high TyG index substantially increases cerebrovascular risk, with a risk ratio of 1.22. A mere 1 mg/dl increase in the TyG index led to a 19.1-fold increase in disease risk [18]. This pattern was observed in a study involving 955 older stroke patients from the Nanjing Stroke Registry in China, where the TyG index emerged as a crucial predictor of stroke recurrence [13]. Additionally, the TyG index has proven its predictive value in rural China, demonstrating its ability to predict ischemic stroke risk [19]. Furthermore, an elevated TyG index has emerged as a risk factor not only for cerebrovascular complications but also for cognitive impairment and cerebral small vessel disease burden [20]. Our study aligns with existing research suggesting that high-fat meals exacerbate systemic free radicals by elevating triglyceride and glucose levels, consequently triggering acute cerebrovascular dysfunction. Conversely, interventions, such as metformin and pitavastatin, ameliorated cerebrovascular risk factors by reducing glucose concentration and triglyceride accumulation [21, 22], as corroborated by our results.

While numerous clinical studies have explored the link between TyG index and stroke as well as atherosclerotic diseases, the underlying mechanisms remain unclear. The TyG index hinges on two crucial components, triglyceride and glucose metabolism, which are markers of IR. This resistance, in turn, increases the risk of chronic metabolic diseases, such as diabetes [23], hypertension [24], and hyperlipidemia [25]. IR is associated with chronic inflammation [26], oxidative stress [27, 28], and endothelial dysfunction [29] and mechanistically paves the way for arterial stenosis [30] and occlusion [31, 32]. Additionally, it influences platelet adhesion [33], activation, and aggregation [34], which are pivotal elements in the pathogenesis of hemodynamic disturbances [35–37]. The TyG index is a composite of fasting triglyceride and fasting glucose levels. The rationale behind the first use of the TyG index in 2008 was that IR is a common cause of elevated triglyceride and glucose levels in healthy individuals, caused by reduced sensitivity and responsiveness to insulin. Many studies have confirmed that IR is associated with lipid abnormalities, hyperglycemia, and hypertension, which cause inflammation, oxidative stress, and free radical production, leading to endothelial damage and smooth muscle dysfunction [38].

Despite the wealth of literature on the association between TyG index and ischemic stroke [39, 40], atherosclerosis [41], and coronary syndromes [42], scant attention has been paid to its connection with CVDs. Our study bridges this gap by establishing a linear association between the TyG index and 28- and 90-day mortality due to CVD. Notably, for every 1 mg/dl increase in TyG, the risk increased by 16% and 18% for 28-day and 90-day mortality, respectively. In the Q4 TyG subgroup, this risk surged to 30% for 28-day and 40% for 90-day mortality. This nuanced understanding provides clinicians with a potent tool for assessing patients with CVD. Moreover, considering the low-cost and widespread availability of triglyceride and glucose tests, these indicators offer a practical and economical approach, significantly reducing healthcare costs and patient burden [43].

Notably, our subgroup analysis revealed a significant difference for patients with CVD. For diabetic outcomes, non-diabetic patients exhibited a higher risk. One study reported a 1.7% increase in the risk of developing diabetes for every unit increase in TyG-body mass index (BMI) < 23.66, whereas, for every unit increase in TyG-BMI > 23.66, there was a 0.7% increase in risk. TyG is attenuated in the presence of diabetes complications [44]. In many studies, the relationship between TyG and diabetes has been explained in terms of IR, a set of symptoms (hyperglycemia, hyperlipidemia, and hypertension) produced by metabolic disorders that lead to an increased risk of disease. Overall, the TyG index has emerged as a valuable clinical tool; however, it is equally crucial to consider other patient-specific factors for a more comprehensive health assessment and tailored treatment strategies in clinical practice.

This study had some limitations. First, because this observational study was analyzed retrospectively, the potential for reverse causality effects cannot be disregarded. Second, although single-center studies utilize a substantial sample size, they may be susceptible to unaccounted potential confounders. Multi-center and multi-ethnic studies would provide more rigorous and generalizable conclusions. Third, the absence of data on human characteristics, such as height and hip-to-waist ratio, from the MIMIC database limits our ability to consider these as potential confounding factors. Fourth, our focus on the initial ICU admission value of the TyG overlooked its dynamics, which might provide further insights. Prospective studies are warranted to validate and reinforce our findings.

## Conclusion

Our study highlights a positive correlation between the TyG index and CVD mortality. This association was notably stronger among patients of varying ages and those without diabetes mellitus. The TyG index has emerged as a valuable predictive tool and robust stratification factor for individuals with CVD. To validate and expand our findings, it is imperative to conduct future prospective trials. These trials will help substantiate the significance of our findings and their practical implications.

### Electronic supplementary material

Below is the link to the electronic supplementary material.

Supplementary Material 1

## Data Availability

Data supporting the findings of this study are available from the Massachusetts Institute of Technology (MIT) and Beth Israel Deaconess Medical Center (BIDMC); however, limitations apply to the availability of these data, which were used with permission for the current study and are therefore not publicly available. However, the data are available to the authors upon reasonable request and with permission from MIT and BIDMC.

## Abbreviations

CVD: Cerebrovascular disease
MIMIC-IV: Medical information mart for intensive care IV
TyG: Triglyceride glucose index
HR: Hazards ratio
CI: Confidence interval
IR: Insulin resistance
SQL: Structured query language
PT: Prothrombin time
PTT: Partial thromboplastin time
WBC: White blood cell count
RBC: Red blood cell
SBP: Systolic blood pressure
DBP: Diastolic blood pressure
SOFA: Sequential organ failure assessment
SD: Standard deviation
IQR: Interquartile range
BMI: Body mass index
MIT: Massachusetts institute of technology
BIDMC: Beth Israel Deaconess medical center
NIH: National institutes of health

## References

[1] Gorelick PB (1986). Cerebrovascular Disease. Pathophysiology and diagnosis. Nurs Clin North Am.

[2] Wolf PA, Grotta JC (2000). Cerebrovascular Disease. Circulation.

[3] Sergi D, Zauli E, Tisato V, Secchiero P, Zauli G, Cervellati C. Lipids at the Nexus between Cerebrovascular Disease and Vascular Dementia: the impact of HDL-Cholesterol and Ceramides. Int J Mol Sci. 2023;24(5). 10.3390/ijms24054403 PMC10002119 36901834

[4] Liu W, Wong A, Law AC, Mok VC (2015). Cerebrovascular Disease, amyloid plaques, and Dementia. Stroke.

[5] Caprio FZ, Sorond FA (2019). Cerebrovascular Disease: primary and secondary Stroke Prevention. Med Clin North Am.

[6] Charidimou A, Werring DJ (2012). Cerebral microbleeds and cognition in Cerebrovascular Disease: an update. J Neurol Sci.

[7] Chalouhi N, Hoh BL, Hasan D (2013). Review of cerebral Aneurysm formation, growth, and rupture. Stroke.

[8] Segura T, Serena J, Castellanos M, Teruel J, Vilar C, Dávalos A (2001). Embolism in acute middle cerebral artery stenosis. Neurology.

[9] MacKenzie JM (1996). Intracerebral haemorrhage. J Clin Pathol.

[10] Ramdas Nayak VK, Satheesh P, Shenoy MT, Kalra S (2022). Triglyceride glucose (TyG) index: a surrogate biomarker of insulin resistance. J Pak Med Assoc.

[11] Sajdeya O, Beran A, Mhanna M, Alharbi A, Burmeister C, Abuhelwa Z (2022). Triglyceride glucose index for the prediction of subclinical Atherosclerosis and arterial stiffness: a Meta-analysis of 37,780 individuals. Curr Probl Cardiol.

[12] Tahapary DL, Pratisthita LB, Fitri NA, Marcella C, Wafa S, Kurniawan F (2022). Challenges in the diagnosis of insulin resistance: focusing on the role of HOMA-IR and Tryglyceride/glucose index. Diabetes Metab Syndr.

[13] Wang F, Wang J, Han Y, Shi X, Xu X, Hou C (2022). Triglyceride-glucose index and Stroke recurrence in elderly patients with ischemic Stroke. Front Endocrinol.

[14] Baydar O, Kilic A, Okcuoglu J, Apaydin Z, Can MM (2021). The triglyceride-glucose index, a predictor of insulin resistance, is Associated with subclinical Atherosclerosis. Angiology.

[15] Wang A, Wang G, Liu Q, Zuo Y, Chen S, Tao B (2021). Triglyceride-glucose index and the risk of Stroke and its subtypes in the general population: an 11-year follow-up. Cardiovasc Diabetol.

[16] Yu Y, Gu M, Huang H, Cheng S, Deng Y, Cai C (2022). Combined association of triglyceride-glucose index and systolic blood pressure with all-cause and cardiovascular mortality among the general population. J Translational Med.

[17] Zhao S, Yu S, Chi C, Fan X, Tang J, Ji H (2019). Association between macro- and microvascular damage and the triglyceride glucose index in community-dwelling elderly individuals: the Northern Shanghai Study. Cardiovasc Diabetol.

[18] Yan F, Yan S, Wang J, Cui Y, Chen F, Fang F (2022). Association between triglyceride glucose index and risk of Cerebrovascular Disease: systematic review and meta-analysis. Cardiovasc Diabetol.

[19] Zhao Y, Sun H, Zhang W, Xi Y, Shi X, Yang Y (2021). Elevated triglyceride-glucose index predicts risk of incident ischaemic Stroke: the rural Chinese cohort study. Diabetes Metab.

[20] Teng Z, Feng J, Dong Y, Xu J, Jiang X, Chen H (2022). Triglyceride glucose index is associated with cerebral small vessel Disease burden and cognitive impairment in elderly patients with type 2 Diabetes Mellitus. Front Endocrinol.

[21] Ishihara Y, Ohmori K, Mizukawa M, Hasan AU, Noma T, Kohno M (2010). Beneficial direct adipotropic actions of pitavastatin in vitro and their manifestations in obese mice. Atherosclerosis.

[22] Xu L, Wang W, Song W (2022). A combination of metformin and insulin improve cardiovascular and cerebrovascular risk factors in individuals with type 1 Diabetes Mellitus. Diabetes Res Clin Pract.

[23] Sims EK, Carr ALJ, Oram RA, DiMeglio LA, Evans-Molina C (2021). 100 years of insulin: celebrating the past, present and future of Diabetes therapy. Nat Med.

[24] Ohishi M (2018). Hypertension with Diabetes Mellitus: physiology and pathology. Hypertens Research: Official J Japanese Soc Hypertens.

[25] Matafome P, Nunes E, Louro T, Amaral C, Crisóstomo J, Rodrigues L (2009). A role for atorvastatin and insulin combination in protecting from liver injury in a model of type 2 Diabetes with hyperlipidemia. Naunyn Schmiedebergs Arch Pharmacol.

[26] Olefsky JM, Glass CK (2010). Macrophages, inflammation, and insulin resistance. Annu Rev Physiol.

[27] Ding X, Jian T, Wu Y, Zuo Y, Li J, Lv H, Biomedicine (2019). & Pharmacotherapy = Biomedecine & Pharmacotherapie.

[28] Yaribeygi H, Farrokhi FR, Butler AE, Sahebkar A (2019). Insulin resistance: review of the underlying molecular mechanisms. J Cell Physiol.

[29] Janus A, Szahidewicz-Krupska E, Mazur G, Doroszko A (2016). Insulin Resistance and endothelial dysfunction constitute a common therapeutic target in Cardiometabolic disorders. Mediators Inflamm.

[30] Diener HC, Hankey GJ (2020). Primary and secondary Prevention of ischemic Stroke and Cerebral Hemorrhage: JACC Focus Seminar. J Am Coll Cardiol.

[31] Painter TA (1991). Myointimal hyperplasia: pathogenesis and implications. 1. In vitro characteristics. Artif Organs.

[32] Aoyagi M, Fukai N, Sakamoto H, Shinkai T, Matsushima Y, Yamamoto M (1991). Altered cellular responses to serum mitogens, including platelet-derived growth factor, in cultured smooth muscle cells derived from arteries of patients with moyamoya Disease. J Cell Physiol.

[33] Brown E, Ozawa K, Moccetti F, Vinson A, Hodovan J, Nguyen TA (2021). Arterial platelet adhesion in atherosclerosis-prone arteries of obese, insulin-resistant nonhuman Primates. J Am Heart Assoc.

[34] Suslova TE, Sitozhevskii AV, Ogurkova ON, Kravchenko ES, Kologrivova IV, Anfinogenova Y (2014). Platelet hemostasis in patients with metabolic syndrome and type 2 Diabetes Mellitus: cGMP- and NO-dependent mechanisms in the insulin-mediated platelet aggregation. Front Physiol.

[35] Kain K, Catto AJ, Grant PJ (2003). Associations between insulin resistance and thrombotic risk factors in high-risk south Asian subjects. Diabet Med.

[36] Kain K, Catto AJ, Young J, Bamford J, Bavington J, Grant PJ (2002). Increased fibrinogen, Von Willebrand factor and tissue plasminogen activator levels in insulin resistant south Asian patients with ischaemic Stroke. Atherosclerosis.

[37] Rusinek H, Ha J, Yau PL, Storey P, Tirsi A, Tsui WH (2015). Cerebral perfusion in insulin resistance and type 2 Diabetes. J Cereb Blood Flow Metab.

[38] Tao LC, Xu JN, Wang TT, Hua F, Li JJ (2022). Triglyceride-glucose index as a marker in Cardiovascular Diseases: landscape and limitations. Cardiovasc Diabetol.

[39] Liao C, Xu H, Jin T, Xu K, Xu Z, Zhu L (2022). Triglyceride-glucose index and the incidence of Stroke: a meta-analysis of cohort studies. Front Neurol.

[40] Zhou Y, Pan Y, Yan H, Wang Y, Li Z, Zhao X (2020). Triglyceride glucose index and prognosis of patients with ischemic Stroke. Front Neurol.

[41] Wu Z, Wang J, Li Z, Han Z, Miao X, Liu X (2021). Triglyceride glucose index and carotid Atherosclerosis incidence in the Chinese population: a prospective cohort study. Nutr Metab Cardiovasc Dis.

[42] Akbar MR, Pranata R, Wibowo A, Irvan, Sihite TA, Martha JW (2021). The association between triglyceride-glucose index and major adverse cardiovascular events in patients with acute coronary syndrome - dose-response meta-analysis. Nutr Metab Cardiovasc Dis.

[43] Li S, Guo B, Chen H, Shi Z, Li Y, Tian Q (2019). The role of the triglyceride (triacylglycerol) glucose index in the development of cardiovascular events: a retrospective cohort analysis. Sci Rep.

[44] Han Y, Hu H, Li Q, Deng Z, Liu D (2023). Triglyceride glucose-body mass index and the risk of progression to Diabetes from prediabetes: a 5-year cohort study in Chinese adults. Front Public Health.

