# Supplementary material for: Association between triglyceride glucose index and all-cause mortality in patients with cerebrovascular disease: a retrospective study
Source: Diabetol Metab Syndr. 2024 Jan 3;16:1. doi: 10.1186/s13098-023-01243-2 (PMC10763128; doi:10.1186/s13098-023-01243-2)
Supplement: Supplementary file 1 — Supplementary Material 1 [file 13098_2023_1243_MOESM1_ESM.docx]

**Table S1**. Missing patients recruited from the MIMIC-IV database.

| **Variable** | **Missing(n)** | **Complete(n)** | **percent_complete(%)** | **percent_missing(%)** |
| --- | --- | --- | --- | --- |
| **Admission age** | 0 | 1965 | 100 | 0 |
| **Antihyperglycemic** | 0 | 1965 | 100 | 0 |
| **Antihyperlipidemic** | 0 | 1965 | 100 | 0 |
| **Atrial_fibrillation** | 26 | 1939 | 98.67684 | 1.323155 |
| **Chloride** | 8 | 1957 | 99.59288 | 0.407125 |
| **Congestive heart failure** | 26 | 1939 | 98.67684 | 1.323155 |
| **Dbp** | 4 | 1961 | 99.79644 | 0.203562 |
| **Dementia** | 26 | 1939 | 98.67684 | 1.323155 |
| **Diabetes** | 26 | 1939 | 98.67684 | 1.323155 |
| **Ethnicity** | 0 | 1965 | 100 | 0 |
| **weight** | 302 | 1663 | 84.63104 | 15.36896 |
| **Glucose** | 8 | 1957 | 99.59288 | 0.407125 |
| **Heart rate** | 3 | 1962 | 99.84733 | 0.152672 |
| **Inr** | 60 | 1905 | 96.94656 | 3.053435 |
| **Los_hospital1** | 0 | 1965 | 100 | 0 |
| **Los_icu1** | 0 | 1965 | 100 | 0 |
| **Mortality_28d** | 0 | 1965 | 100 | 0 |
| **Mortality_365d** | 0 | 1965 | 100 | 0 |
| **Mortality_90d** | 0 | 1965 | 100 | 0 |
| **Myocardial infarct** | 26 | 1939 | 98.67684 | 1.323155 |
| **Paraplegia** | 26 | 1939 | 98.67684 | 1.323155 |
| **Peripheral vascular disease** | 26 | 1939 | 98.67684 | 1.323155 |
| **Platelet** | 4 | 1961 | 99.79644 | 0.203562 |
| **Pt** | 60 | 1905 | 96.94656 | 3.053435 |
| **Ptt** | 66 | 1899 | 96.64122 | 3.358779 |
| **Rbc** | 3 | 1962 | 99.84733 | 0.152672 |
| **Renal disease** | 26 | 1939 | 98.67684 | 1.323155 |
| **Respiratory rate** | 7 | 1958 | 99.64377 | 0.356234 |
| **Sbp** | 4 | 1961 | 99.79644 | 0.203562 |
| **Sex** | 0 | 1965 | 100 | 0 |
| **Sofa** | 0 | 1965 | 100 | 0 |
| **Triglycerides** | 0 | 1965 | 100 | 0 |
| **WBC** | 2 | 1963 | 99.89822 | 0.101781 |

TyG, triglyceride glucose index; Inr, international normalized ratio; Pt, Prothrombin time; Ptt, Partial Thromboplastin Time; WBC, white blood cell; RBC, red blood cell;Sbp, systolic blood pressure; Dbp, diastolic blood pressure; SOFA; sequential organ failure assessment.

**Table S2**. Patients' in-hospital and ICU survival times and mortality rates

| **TYG quartile** | **Overall** | **Q1** | **Q2** | **Q3** | **Q4** | ***p*-value** |
| --- | --- | --- | --- | --- | --- | --- |
| **N** | 1,965, | 491 | 491 | 491 | 492 |  |
| **Los hospital(days,IQR)** | 6.83(3.92-12.95) | 6.27 (3.85-10.75) | 6.70 (3.79-12.57) | 6.97 (4.08-13.93) | 7.81 (4.12-15.96) | <0.001 |
| **Los icu(days,IQR)** | 3.01(1.81-5.84) | 2.77 (1.79-4.88) | 3.00 (1.84-5.63) | 3.27 (1.83-6.50) | 3.21 (1.78-7.16) | 0.003 |
| **Mortality_28d** |  |  |  |  |  | 0.098 |
| **Alive** | 1570(79.90%) | 409 (83.30%) | 386 (78.62%) | 395 (80.45%) | 380 (77.24%) |  |
| **Dead** | 395(20.10%) | 82 (16.70%) | 105 (21.38%) | 96 (19.55%) | 112 (22.76%) |  |
| **Mortality_90d** |  |  |  |  |  | 0.027 |
| **Alive** | 1484(75.52%) | 391 (79.63%) | 362 (73.73%) | 377 (76.78%) | 354 (71.95%) |  |
| **Dead** | 481(24.48%) | 100 (20.37%) | 129 (26.27%) | 114 (23.22%) | 138 (28.05%) |  |

TyG, triglyceride glucose index. IQR, interquartile range. TyG index quartiles: Q1 (7.21–8.40)mg/dl, Q2 (8.40–8.80) mg/dl, Q3 (8.80–9.24) mg/dl, Q4 (9.24–12.96) mg/dl.

**Table S3.** Predictive efficacy of TyG and respective triglycerides and glucose.

| **Variable** | **Cut.value** | **Sensitivity** | **Specificity** | **AUC** |
| --- | --- | --- | --- | --- |
| **28-day mortality** |  |  |  |  |
| **TyG** | 0.016 | 0.392 | 0.696 | 0.540 |
| **Triglyceride + Glucose** | 4e-05 | 0.431 | 0.702 | 0.571 |
| **90-day Mortality** |  |  |  |  |
| **TyG** | 0.013 | 0.362 | 0.688 | 0.514 |
| **Triglyceride + Glucose** | 0.006 | 0.372 | 0.730 | 0.542 |

TyG, triglyceride glucose index; AUC, area under curve.

**Table S4.** For cox proportional risk ratios that exclude the use of antihyperglycemic.

| **Exposure** | **Model I^a^**  **HR (95%CI) *p*** | ***p* for trend** | **Model II^b^**  **HR (95%CI) *p*** | ***p* for trend** | **Model III^c^**  **HR (95%CI) *p*** | ***p* for trend** | **Model IV^d^**  **HR (95%CI) *p*** | ***p* for trend** |
| --- | --- | --- | --- | --- | --- | --- | --- | --- |
| **Mortality_28d** |  |  |  |  |  |  |  |  |
| **TYG** | 0.94 (0.82, 1.08) 0.3980 |  | 1.16 (1.01, 1.33) 0.0388 |  | 1.15 (0.99, 1.35) 0.0762 |  | 1.16 (0.99, 1.37) 0.0601 |  |
| **TYG quartile** |  | 0.497 |  | 0.035 |  | 0.099 |  | 0.09 |
| **Q1** | Ref |  | Ref |  | Ref |  | Ref |  |
| **Q2** | 1.14 (0.85, 1.52) 0.3826 |  | 1.22 (0.91, 1.63) 0.1795 |  | 1.16 (0.86, 1.57) 0.3181 |  | 1.16 (0.86, 1.56) 0.3364 |  |
| **Q3** | 0.92 (0.68, 1.24) 0.5861 |  | 1.16 (0.86, 1.57) 0.3249 |  | 1.13 (0.83, 1.54) 0.4395 |  | 1.13 (0.83, 1.55) 0.4282 |  |
| **Q4** | 0.97 (0.73, 1.30) 0.8613 |  | 1.42 (1.06, 1.91) 0.0199 |  | 1.36 (0.98, 1.88) 0.0683 |  | 1.36 (0.98, 1.89) 0.0637 |  |
| **Mortality_90d** |  |  |  |  |  |  |  |  |
| **TYG** | 0.93 (0.83, 1.06) 0.2816 |  | 1.15 (1.01, 1.30) 0.0306 |  | 1.17 (1.02, 1.36) 0.0283 |  | 1.19 (1.03, 1.37) 0.0206 |  |
| **TYG quartile** |  | 0.355 |  | 0.035 |  | 0.0577 |  | 0.049 |
| **Q1** | Ref |  | Ref |  | Ref |  | Ref |  |
| **Q2** | 1.16 (0.89, 1.51) 0.2663 |  | 1.23 (0.95, 1.61) 0.1179 |  | 1.19 (0.91, 1.56) 0.2065 |  | 1.18 (0.90, 1.55) 0.2280 |  |
| **Q3** | 0.90 (0.69, 1.18) 0.4397 |  | 1.13 (0.86, 1.49) 0.3691 |  | 1.11 (0.84, 1.48) 0.4700 |  | 1.11 (0.84, 1.48) 0.4628 |  |
| **Q4** | 0.97 (0.74, 1.25) 0.7938 |  | 1.39 (1.07, 1.82) 0.0152 |  | 1.39 (1.03, 1.87) 0.0287 |  | 1.40 (1.04, 1.88) 0.0256 |  |

TyG, triglyceride glucose index; HR, Harzard ratio;95% CI,95% confidence interval.

^a^ was unadjusted.

^b^was adjusted for Sex, admission Age, Weight and Ethnicity.

^c^was adjusted for Sex, admission Age, Weight and Ethnicity, INR, PT, PTT, WBC, Chloride, RBC, Platelet, Heart Rate, Respiratory Rate, Sbp, Dbp, myocardial infarct, congestive heart failure, peripheral vascular disease, dementia, paraplegia, renal disease, atrial fibrillation, diabetes and sofa.

^d^was adjusted for Sex, admission Age, Weight and Ethnicity, INR, PT, PTT, WBC, Chloride, RBC, Platelet, Heart Rate, Respiratory Rate, Sbp, Dbp, myocardial infarct, congestive heart failure, peripheral vascular disease, dementia, paraplegia, renal disease, atrial fibrillation, diabetes, sofa and Antihyperlipidemic.

TyG index quartiles: Q1 (7.21–8.40)mg/dl, Q2 (8.40–8.80) mg/dl, Q3 (8.80–9.24) mg/dl, Q4 (9.24–12.96) mg/dl.

**Table S5.** For cox proportional risk ratios that exclude the use of antihyperlipidemic.

| **Exposure** | **Model I^a^**  **HR (95%CI) *p*** | ***p* for trend** | **Model II^b^**  **HR (95%CI) *p*** | ***p* for trend** | **Model III^c^**  **HR (95%CI) *p*** | ***p* for trend** | **Model IV^d^**  **HR (95%CI) *p*** | ***p* for trend** |
| --- | --- | --- | --- | --- | --- | --- | --- | --- |
| **Mortality_28d** |  |  |  |  |  |  |  |  |
| **TYG** | 1.08 (0.92, 1.27) 0.3592 |  | 1.37 (1.15, 1.63) 0.0005 |  | 1.30 (1.07, 1.58) 0.0090 |  | 1.28 (1.05, 1.55) 0.0158 |  |
| **TYG quartile** |  | 0.338 |  | 0.002 |  | 0.018 |  | 0.028 |
| **Q1** | Ref |  | Ref |  | Ref |  | Ref |  |
| **Q2** | 1.05 (0.72, 1.52) 0.8017 |  | 1.17 (0.80, 1.69) 0.4177 |  | 1.07 (0.72, 1.57) 0.7493 |  | 1.03 (0.69, 1.52) 0.8914 |  |
| **Q3** | 0.96 (0.66, 1.38) 0.8162 |  | 1.23 (0.84, 1.78) 0.2875 |  | 1.18 (0.81, 1.74) 0.3894 |  | 1.14 (0.77, 1.68) 0.5054 |  |
| **Q4** | 1.21 (0.85, 1.72) 0.2866 |  | 1.75 (1.22, 2.50) 0.0024 |  | 1.58 (1.07, 2.34) 0.0214 |  | 1.53 (1.03, 2.27) 0.0350 |  |
| **Mortality_90d** |  |  |  |  |  |  |  |  |
| **TYG** | 1.06 (0.91, 1.24) 0.4226 |  | 1.36 (1.16, 1.60) 0.0002 |  | 1.32 (1.10, 1.58) 0.0025 |  | 1.30 (1.09, 1.56) 0.0044 |  |
| **TYG quartile** |  |  |  |  |  |  |  |  |
| **Q1** | Ref | 0.514 | Ref | 0.002 | Ref | 0.014 | Ref | 0.02 |
| **Q2** | 1.03 (0.74, 1.44) 0.8483 |  | 1.14 (0.81, 1.59) 0.4569 |  | 1.03 (0.72, 1.46) 0.8820 |  | 0.99 (0.70, 1.42) 0.9752 |  |
| **Q3** | 0.91 (0.65, 1.28) 0.5871 |  | 1.17 (0.83, 1.65) 0.3633 |  | 1.13 (0.79, 1.60) 0.5070 |  | 1.09 (0.76, 1.55) 0.6336 |  |
| **Q4** | 1.14 (0.83, 1.57) 0.4124 |  | 1.66 (1.19, 2.30) 0.0025 |  | 1.56 (1.09, 2.23) 0.0157 |  | 1.51 (1.06, 2.17) 0.0242 |  |

TyG, triglyceride glucose index; HR, Harzard ratio;95% CI,95% confidence interval.

^a^ was unadjusted.

^b^was adjusted for Sex, admission Age, Weight and Ethnicity.

^c^was adjusted for Sex, admission Age, Weight and Ethnicity, INR, PT, PTT, WBC, Chloride, RBC, Platelet, Heart Rate, Respiratory Rate, Sbp, Dbp, myocardial infarct, congestive heart failure, peripheral vascular disease, dementia, paraplegia, renal disease, atrial fibrillation, diabetes and sofa.

^d^was adjusted for Sex, admission Age, Weight and Ethnicity, INR, PT, PTT, WBC, Chloride, RBC, Platelet, Heart Rate, Respiratory Rate, Sbp, Dbp, myocardial infarct, congestive heart failure, peripheral vascular disease, dementia, paraplegia, renal disease, atrial fibrillation, diabetes, sofa and antihyperglycemic.

IQR, interquartile range. TyG index quartiles: Q1 (7.21–8.40)mg/dl, Q2 (8.40–8.80) mg/dl, Q3 (8.80–9.24) mg/dl, Q4 (9.24–12.96) mg/dl.
